# Bilateral Knee Extensor Fatigue Modulates Force and Responsiveness of the Corticospinal Pathway in the Non-fatigued, Dominant Elbow Flexors

**DOI:** 10.3389/fnhum.2016.00018

**Published:** 2016-02-01

**Authors:** Nemanja Šambaher, Saied Jalal Aboodarda, David George Behm

**Affiliations:** School of Human Kinetics and Recreation, Memorial University of Newfoundland, St. John’sNL, Canada

**Keywords:** crossover fatigue, non-local muscle fatigue, cross-education, electromyography, corticospinal excitability, fatigue

## Abstract

Exercise-induced fatigue affects muscle performance and modulates corticospinal excitability in non-exercised muscles. The purpose of this study was to investigate the effect of bilateral knee extensor fatigue on dominant elbow flexor (EF) maximal voluntary force production and corticospinal excitability. Transcranial magnetic, transmastoid electrical and brachial plexus electrical stimulation (BPES) were used to investigate corticospinal, spinal, and muscle excitability of the dominant EF before and after a bilateral knee extensor fatiguing protocol or time matched rest period (control). For both sessions three stimuli were delivered every 1.5 s during the three pre-test time points and during the 1st, 3rd, 6th, 9th and 12th post-test 5 s EF isometric maximal voluntary contractions (MVC). In both conditions, overall, EF MVC force (*p* < 0.001) decreased progressively from repetition #1 to #12 during the post-test MVC protocol. EF MVC force (*p* < 0.001, *ES* = 0.9, Δ10.3%) decrements were more pronounced in the knee extensor fatigue intervention condition. In addition, there were no significant differences between conditions for biceps brachii electromyographic (EMG) activity (*p* = 0.43), motor evoked potentials (MEPs) amplitude (*p* = 0.908) or MEP silent period (SP; *p* = 0.776). However, the fatigue condition exhibited a lower MEP/cervicomedullary MEP (CMEP) ratio (*p* = 0.042, *ES* = 2.5, Δ25%) and a trend toward higher CMEP values (*p* = 0.08, *ES* = 0.5, Δ20.4%). These findings suggest that bilateral knee extensor fatigue can impair performance and modulate corticospinal excitability of the EF.

## Introduction

Exercise-induced neuromuscular fatigue can reduce maximal force output and corticospinal excitability in exercised muscles (Gandevia, 2001). Fatiguing contractions can also alter cortico-motor responses (Brasil-Neto et al., 1993; Todd et al., 2003b; Takahashi et al., 2011; Sidhu et al., 2014) and muscle performance (Halperin et al., 2014a,b) in non-exercised muscles. Fatigue in one muscle group, which leads to an acute drop in muscle performance in another muscle group, has been termed non-local muscle fatigue (NLMF; Zijdewind et al., 1998). This phenomenon has been demonstrated for heteronymous (Kennedy et al., 2013a; Halperin et al., 2014a,b) and homonymous muscles (Martin and Rattey, 2007; Halperin et al., 2014b; Kawamoto et al., 2014). Studies which exhibited force and voluntary activation decrements in the rested non-local muscle groups have also demonstrated an absence of peripheral fatigue (Rattey et al., 2006; Martin and Rattey, 2007; Kennedy et al., 2013a), which strongly supports the hypothesis that central mechanisms contribute to the NLMF phenomenon.

Fatiguing contractions can alter the metabolic environment in the working muscles, thus leading to an increase in the discharge rate of group III and IV muscle afferents (Amann, 2012; Amann et al., 2013; Kennedy et al., 2015). These afferents react to increases in ATP, lactate, and H+ concentration (Kennedy et al., 2015), which through a feedback loop provide an inhibitory input to the central nervous system (CNS; Amann, 2012; Amann et al., 2013). It is thought that this inhibition can cause a reduction in voluntary activation and maximal force output of the non-exercised muscles (Amann et al., 2013; Kennedy et al., 2013b, 2014). Sidhu et al. (2014) found that a lower body cycling task to failure reduced elbow flexor (EF) maximal voluntary contraction (MVC) and voluntary activation and suggested that group III/IV afferent feedback was responsible for a “spill-over” of central fatigue. On the other hand, Kennedy et al. (2013a) found that maximal and submaximal handgrip contractions to task failure affected ankle plantar flexor MVC and voluntary activation. Similarly, Halperin et al. (2014b) found a significant decrease in force (8%) and voluntary activation (5.5%) of knee extensors following a unilateral EF isometric fatiguing protocol. Although these findings advocated that the exercise-induced fatigue in one extremity (e.g., knee extensors) could impair muscle performance in another extremity (e.g., EFs), few studies have investigated the responsiveness of supraspinal and spinal circuitries supplying central commands to the non-fatigued EFs.

Using transcranial magnetic stimulation (TMS), Sidhu et al. (2014) demonstrated a decrease in the amplitude of motor evoked potentials (MEPs) recorded from the EFs following lower body cycling task to failure. Takahashi et al. (2011) also demonstrated that a leg press fatiguing protocol could affect corticospinal excitability to the first dorsal interosseus and biceps brachii muscles. However, MEP amplitude used in these two studies was a measure of corticospinal excitability, therefore it is not known how spinal motoneuron excitability would contribute to corticospinal responses recorded from non-exercised limb. Evidence regarding the role of spinal motoneuron excitability in modulation of central motor drive to non-exercised muscles in the other extremity is scarce. Aboodarda et al. (2015) demonstrated that bilateral EF fatigue could increase knee extensor spinal motoneuron excitability, however these authors did not measure supraspinal excitability. Therefore, further research is required to elucidate the changes in supraspinal and spinal excitability in the non-exercise EFs following knee extensors muscle fatigue.

In a previous study from our laboratory (Halperin et al., 2014a), we found that bilateral knee extensor fatigue reduced dominant EF maximal force production only in the last five MVCs of the 12 post-test MVC contractions. Since voluntary activation and corticospinal excitability of the EFs were not measured in that study, it was not clear how the supraspinal and spinal structures mediated central command to the non-fatigued EFs. Therefore, by employing a very similar experimental design, we aimed to investigate the muscle performance and corticospinal excitability (stimulation of both motor-cortical and subcortical areas to monitor supraspinal and spinal excitability, respectively) of the EF muscles before and after bilateral knee extensor fatigue.

## Materials and Methods

### Participants

Fourteen healthy male (178 ± 4 cm, 78 ± 6 kg, 24 ± 3 years) active but not specifically trained participants from the university population volunteered for the study. None of the participants had a history of musculoskeletal or neurological disease or were taking medications. Thirteen participants were right hand dominant as determined using the Edinburgh handedness inventory (Veale, 2014). They were verbally informed of the procedures and provided written consent prior to participation. The procedures were conducted in accordance with declaration of Helsinki and approved by the Health Research Ethics Authority of Memorial University of Newfoundland (#20141100-HK). Prior to study commencement, each participant completed a magnetic stimulation safety questionnaire for potential contraindications with magnetic stimulation procedures (Rossi et al., 2009) and Physical Activity Readiness Questionnaire (Canadian Society for Exercise Physiology, 2003). Participants were asked to refrain from ingesting caffeine or participating in vigorous physical activity at least 1 day before attending each experimental session.

### Experimental Overview

**Figure 1:**
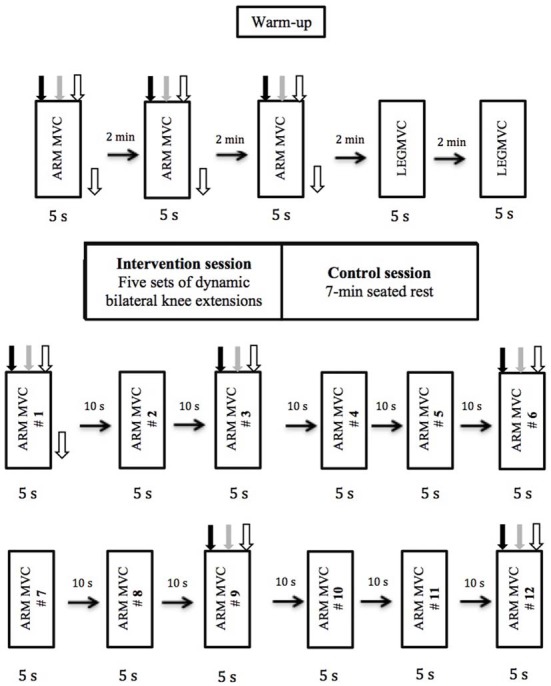
**Experimental design and procedures.** The order in which the different types of stimulations were delivered is depicted and was kept constant for all subjects and for both sessions. Black arrow pointing down represents transcranial magnetic stimulation (TMS), gray arrow represents transmastoid electric stimulation, white arrow represent brachial plexus electrical stimulation (BPES) and black arrow pointing to the right represents rest period.

Subjects attended the laboratory on two occasions separated by at least 5 days and performed one of the two conditions in a random and counterbalanced order (Figure 1): (1) control (no intervention) and (2) intervention (bilateral knee extensor fatigue protocol). Before and after each experimental condition, elbow flexion maximal voluntary isometric contractions (MVCs) were performed and motor-cortical, spinal and muscle responses were recorded from dominant biceps brachii muscle during MVCs.

### Experimental Set Up

At the beginning of each session, participants were equipped with surface electrodes for both stimulation and electromyographic (EMG) activity recording on the dominant arm. EMG was recorded from biceps brachii and triceps brachii (lateral head) muscles using pairs of self-adhesive Ag/AgCl electrodes (Kendall MediTrace foam electrodes, MA, USA) placed 2 cm apart (center to center) on the mid-muscle belly (Hermens et al., 1999). A ground electrode was placed on the lateral epicondyle. Before the placement of electrodes, the area of skin was shaved and abraded to remove dead skin with sandpaper and cleansed with an isopropyl alcohol swab to decrease skin resistance. An inter-electrode impedance of <5 kΩ was obtained prior to recording to ensure an adequate signal-to-noise ratio. EMG electrode locations were highlighted with indelible ink to ensure proper positioning for the subsequent testing session. All EMG signals were recorded (Biopac System Inc., DA 100: analog-digital converter MP150WSW; Holliston, MA, USA) with a sampling rate of 5000 Hz using a commercially designed software program (AcqKnowledge III, Biopac System Inc.). EMG signals were amplified (×1000, bi-polar differential amplifier, input impedance = 2 MΩ, common mode rejection ratio >110 dB min (50/60 Hz), noise >5 μV), analog-to-digitally converted (12 bit), filtered with 10–500 Hz band-pass filter and stored on personal computer for further analysis.

After 5 min cycling on a stationary bike at a cadence of 70 rpm at 1 kp, participants were seated in the knee extension machine (Modular Leg Extension, Cybex International, Medway, MA, USA) with their upper arm supported and elbow flexed at 90°, and with the hip and knee fixed at 90° and 83°, respectively. The knee flexion angle was pre-determined by the inclined angle of the seat, which could not be adjusted. The dominant wrist and ankle were inserted into a padded straps attached by a high tension wire to a load cell (Omega Engineering Inc., LCCA 500 pounds; sensitivity = 3 mV/V, OEI, Canada) that was used to measure elbow flexion and knee extension force, respectively. To eliminate upper body involvement, a strap was placed around the waist and upper body. Following positioning on the chair, the participants performed a muscle warm-up that included 12 brief (2 s “on” and 2 s “off”) dominant EF contractions at 50% of perceived MVC. Then subjects performed one 5 s isometric elbow flexion MVC with their dominant arm, which was subsequently used to determine 5% MVC during which appropriate stimulation intensities were ascertained for three motor responses recorded from dominant biceps brachii muscle via: (1) TMS; (2) transmastoid electric stimulation (TMES); and (3) brachial plexus electrical stimulation (BPES). Appropriate stimulation intensities were determined for TMS and TMES during a 5% MVC of the EFs as opposed to complete rest, since excitability of the corticospinal tract increases significantly from rest to low intensity contractions (Taylor et al., 1997). Participants received real-time visual feedback regarding the intensity of the elbow flexion from a computer monitor screen.

#### Transcranial Magnetic Stimulation (TMS)

MEP responses of the biceps brachii muscle were elicited using a Magstim 200 stimulator (Magstim Company, UK) with a circular coil (13.5 cm outside diameter) centered at the vertex and oriented tangentially to the scalp in an anterior posterior direction. The current in the coil flowed clockwise (preferential stimulation of the right hemisphere for left-handed participant) and anticlockwise (preferential stimulation of the left hemisphere for right-handed participants). The vertex was marked directly on the scalp by measuring the distance halfway from tragus to tragus and from nasion to inion (Pearcey et al., 2014). The position was marked on the scalp with ink to allow an accurate repositioning of the coil throughout the whole experiment. The TMS intensity was increased stepwise to produce MEP amplitudes of approximately 20% of M-max in the biceps muscle during brief 5% MVC contraction. The mean stimulation intensity was 56 ± 11% of maximum stimulator output. The MEP amplitude recorded at this TMS intensity could be differentiated from the background EMG, during 100% MVC contractions. This stimulation intensity was then used for the remainder of the experiment.

#### Transmastoid Electrical Stimulation (TMES)

The descending corticospinal tract was stimulated at the level of cervicomedullary junction (pyramidal decussation), eliciting cervicomedullary MEP (CMEPs). A high-voltage electrical current was passed between surface electrodes placed over the skin covering mastoid processes stimulator Model DS7AH; Digitimer, Welwyn Garden City, Hertfordshire, UK). The stimulation intensity (pulse duration: 100 μs; 400 volt square-wave) was adjusted to CMEP amplitudes that matched the MEP amplitudes during a brief 5% MVC contraction (Pearcey et al., 2014).

#### Brachial Plexus Electrical Stimulation (BPES)

To determine the size of the maximal compound muscle action potential (M-max) of the biceps brachii, the peripheral nerve innervating EF and extensor muscles was stimulated by a single stimulus elicited at the brachial plexus area called the Erb’s point. The stimulating electrodes (Ag-AgCl discs, 20 mm diameter) were placed on the supraclavicular fossa (cathode) and on the acromion process (anode). BPES was performed using high-voltage percutaneous electrical stimuli (stimulator Model DS7AH; Digitimer, Welwyn Garden City, Hertfordshire, UK). The stimulation intensity (200 μs pulse duration; 400 volt square-wave) increased in incremental steps (20 mA) until a plateau in compound action potential was achieved.

### Experimental Protocol

The experimental protocol (Figure 1) used for this study was very similar to the one used previously in our laboratory (Halperin et al., 2014a). Subjects performed 3-EF MVCs of the dominant arm with 2 min rest between trials. A set of three responses (MEP, CMEP and M-max) were recorded with an inter-stimulus interval of 1.5 s during 5 s MVCs (Pearcey et al., 2014). During each contraction, MEP, CMEP and M-max were elicited at 2, 3.5 and 5 s, respectively. TMS and TMES were triggered automatically, whereas BPES stimulation of the peripheral nerve was performed manually at 5 s. All participants were able to regain voluntary force adequately after each stimulus. The reason that MEP was evoked at 2 s was to give the participants adequate time to reach maximal force production. After completion of the upper-body pretest measurements, subjects performed a warm-up for the knee extensors, consisting of 12 isometric contractions at 50% of perceived knee extension MVC. This was followed by two dominant knee extension MVCs with 2 min rest between contractions. Then, subjects either performed the fatiguing protocol (intervention condition) or rested for 7 min (control condition).

The fatiguing protocol used in this study has been employed previously in our laboratory (Halperin et al., 2014a), which resulted in considerable knee extensor muscle fatigue (i.e., post-test MVC dropped by 35% compared with baseline values) and accumulation of blood lactate. The fatiguing protocol consisted of five sets of dynamic bilateral knee extensor contractions performed until task failure. The load was equivalent to the isometric knee extension MVC force in dominant leg. One-minute rest was given between sets. Failure was defined as the inability to fully extend the knee during the contractions, which was measured by touching the shin to an exercise band tied parallel to the ground at full extension, or by not keeping a constant pace of “1 s concentric and 1 s eccentric contraction” dictated by a metronome. Subjects were constantly verbally motivated during the protocol and were reminded to keep their upper body as relaxed as possible. Biceps brachii activity was monitored throughout the entire fatiguing protocol to ensure that EMG activity was no different than during rest. If there was evidence of activation exceeding 0.05V, the subjects were first reminded to relax their arm, and if after two warnings they were not able to relax their biceps brachii muscle, exercise was stopped. Immediately after the last set of knee extension contractions, subjects performed the post-test protocol, which included 12 isometric elbow flexion MVCs at a work to rest ratio of 5–10 s. During the 1st, 3rd, 6th, 9th and 12th MVC, subjects received the same set of stimuli as the pre-test MVCs. During the control session, subjects underwent the exact same pre- and post-test measurements, but instead of performing the fatiguing dynamic knee extensions, they sat on the knee extension chair for 7 min, which was the approximate time period required to complete the fatiguing protocol.

### Outcome Measures

#### Knee Extensors

During the fatigue session, the number of repetitions performed at each set was measured.

#### Dominant Elbow Flexors

The following variables were measured for the three elbow flexion MVCs at pre-test, as well as the 1st, 3rd, 6th, 9th and 12th post-test MVCs. Mean muscle force production and the background EMG [root mean square (rmsEMG)] of the biceps and triceps brachii muscles were quantified over 500 ms duration prior to the point of each stimulation (TMS, TMES, BPES). Peak-to-peak amplitudes of the MEP, CMEP and M-max were measured. The duration of silent period (SP; ms) was assessed for MEPs as the interval from the stimulus artifact to the return of the continuous EMG by visual inspection. Because the M-max can change as a result of the voluntary activation, the MEP and CMEP were divided by the following M-max during each MVC. Normalized MEP and CMEP data made it possible to compare these values between different testing sessions. The recorded MEP from the target muscle, elicited by magnetic stimulation of the motor cortex, accesses the entire motor pathway from the motor cortex to the muscles performing the task. Stimulation of corticospinal pathway at the transmastoid level evokes CMEP from the same motor axons that are activated by TMS (Gandevia et al., 1999). Therefore, differences between the MEP and CMEP (MEP/CMEP ratio) were measured to assess whether any changes were occurring at the supraspinal or spinal levels.

To account for variability in the outcome measurements between the testing days, all EF dependent variables were normalized to the average value of the three pre-test trials and as such are reported as a percentage.

### Statistical Analysis

Statistical analyses were computed using SPSS software (Version 16.0, SPSS, Inc, Chicago, IL, USA). Assumption of normality (Shapiro-Wilk test) and sphericity (Mauchley test) were tested for all of the dependent variables. If the assumption of sphericity was violated, the corrected value for non-sphericity with Greenhouse-Geisser epsilon was reported. First, intraclass correlation coefficients (ICC) were measured for mean force, and EMG for the three pretests of both conditions to assess consistency of this data. Second, a two-way repeated measures analysis of variance (ANOVA; 2 conditions × 5 MVCs) was conducted to determine differences between conditions in the following variables: normalized dominant elbow flexion MVC force, biceps brachii and triceps brachii EMG, MEP, CMEP and M-max amplitude, MEP/CMEP ratio, and cortical SP. To ensure that background EMG activity was similar before TMS and TMES, we compared EMG activity 500 ms before each stimulus. Paired sample *t*-tests corrected with Bonferroni were used to decompose significant interactions, and Bonferroni *post hoc* tests were used if main effects were found. Significance was set at 0.05. Cohen’s *d* effects sizes (ES; Cohen, 1988) were also calculated to investigate the standardized magnitude of change for all significant results according to the criterion of *d* < 0.2 was classified as “trivial”, *d* = 0.2–0.49 was considered as “small” effect size; *d* = 0.5–0.79 represented a “medium” effect size; and *d* > 0.8 represented a “large” effect size (Cohen, 1988). Data in the text are reported as means ± SD, and shown in the figures as means ± SE.

## Results

### Reliability

The ICC of pre-test trials for various measurements between two testing sessions was as follows: absolute force (0.92), EMG of biceps brachii (0.87) and triceps brachii (0.94).

### Knee Extension Fatigue Protocol

All subjects were able to successfully complete the knee extension fatiguing protocol. The number of repetitions (mean ± SD) decreased throughout the five sets: set 1 (23 ± 6), set 2 (16 ± 3), set 3 (14 ± 3), set 4 (13 ± 3) and set 5 (10 ± 3).

### Elbow Flexion MVC

**Figure 2:**
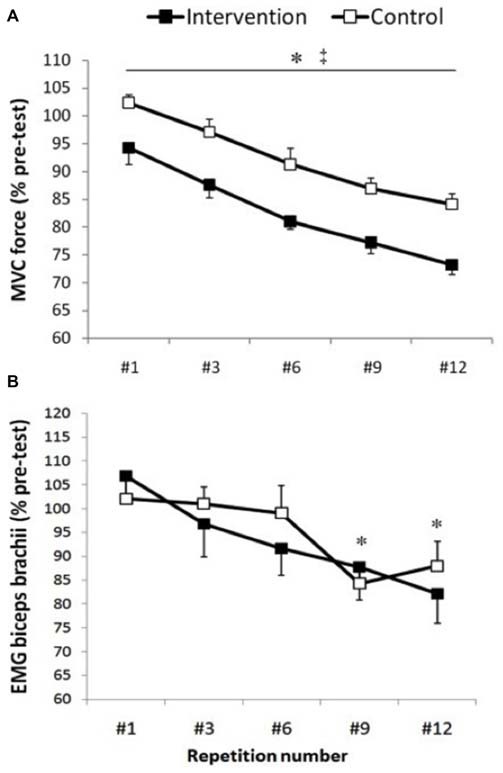
**Maximal voluntary contractions (MVC) force and biceps brachii electromyographic (EMG).** Group data are expressed as means ± SE and normalized to pre-test. **(A)** Arm MVC force normalized to pre-test. ‡Indicates that MVC force was significantly lower in the intervention condition compared with control condition (*p* < 0.001). *Indicates that MVC force significantly and progressively decreases from repetition #1 until repetition #12 (*p* < 0.001). **(B)** EMG activity of the biceps brachii muscle measured 50 ms before motor evoked potentials (MEPs). *Indicates that biceps brachii EMG activity was significantly lower at repetition #9 and #12 compared with repetition #1, #3 and #6 (*p* < 0.04).

Normalized elbow flexion MVC (Figure 2) force showed a main effect for condition (*F*_(1,13)_ = 18.0, *p* < 0.001) and repetitions (*F*_(4,52)_ = 41.77, *p* < 0.001), whereas there was no significant interaction of condition × repetitions (*F*_(4,52)_ = 0.16, *p* = 0.953). The average MVC of all post-test MVCs was significantly lower in the intervention session compared with control session (*p* < 0.001, *ES* = 0.9, *Δ* = 10.3%; Table 1). Furthermore, the maximal force progressively and significantly decreased from repetition #1 to repetition #12 (*p* < 0.001; Figure 2). Repetition #1 was significantly higher compared with repetition #3 (*p* = 0.003, *ES* = 0.6, Δ6%), repetition #6 (*p* < 0.001, *ES* = 1.2, Δ12.1%), repetition #9 (*p* < 0.001, *ES* = 1.7, Δ16.2%) and repetition #12 (*p* < 0.001, *ES* = 2.1, Δ19.6%).

**Table 1:** **The mean (±*SD*) of the absolute data recorded from the non-exercised dominant elbow flexors muscles including compound muscle action potential (M-max) and motor evoked potentials (MEPs) and cervicomedullary evoked potentials (CMEP) normalized to M-max recorded from non-exercised biceps brachii muscle, MEP/CMEP ratio, duration of MEP silent period (SP) for two conditions (Intervention and Control) and five time points (repetition number 1, 3, 6, 9 and 12)**.

Variable	Conditions	Pre-test	Repetition #1	Repetition #3	Repetition #6	Repetition #9	Repetition #12
MVC (N)	Intervention	375.8 (59)	352.8 (64)	327.6 (53)	304.3 (49)	288.8 (45)	275.2 (49)
	Control	358.6 (46)	366.9 (50)	347.2 (49)	323.7 (30)	309.8 (33)	300.6 (40)
Biceps EMG (mV)	Intervention	0.45 (0.17)	0.50 (0.18)	0.44 (0.14)	0.42 (0.13)	0.40 (0.14)	0.37 (0.12)
	Control	0.36 (0.10)	0.37 (0.13)	0.37 (0.10)	0.36 (0.10)	0.31 (0.08)	0.31 (0.07)
M-max	Intervention	5.68 (1.80)	6.21 (2.04)	6.79 (2.35)	7.02 (2.25)	6.96 (2.18)	6.72 (2.70)
	Control	5.42 (1.44)	5.51 (1.81)	5.76 (1.65)	5.86 (1.28)	5.84 (1.61)	6.01 (1.34)
MEP/M-max ratio	Intervention	0.67 (0.18)	0.69 (0.19)	0.73 (0.18)	0.69 (0.21)	0.65 (0.16)	0.71 (0.23)
	Control	0.62 (0.11)	0.70 (0.22)	0.68 (0.25)	0.67 (0.21)	0.66 (0.18)	0.59 (0.22)
CMEP/M-max ratio	Intervention	0.55 (0.14)	0.56 (0.19)	0.52 (0.18)	0.71 (0.49)	0.58 (0.28)	0.67 (0.29)
	Control	0.54 (0.14)	0.50 (0.22)	0.53 (0.24)	0.52 (0.19)	0.46 (0.20)	0.40 (0.23)
MEP/CMEP	Intervention	1.35 (0.45)	1.37 (0.63)	1.66 (0.90)	1.14 (0.42)	1.28 (0.58)	1.24 (0.60)
	Control	1.35 (0.30)	1.57 (0.61)	1.41 (0.52)	1.58 (1.07)	1.72 (0.74)	1.86 (0.96)
MEP Silent Period (sec)	Intervention	0.12 (0.03)	0.12 (0.03)	0.11 (0.04)	0.11 (0.04)	0.12 (0.04)	0.11 (0.04)
	Control	0.12 (0.05)	0.12 (0.05)	0.12 (0.05)	0.12 (0.05)	0.13 (0.05)	0.12 (0.05)

### Biceps and Triceps Brachii rmsEMG

Biceps brachii rmsEMG before MEP, CMEP and M-wave showed a significant main effect for repetitions (*p* < 0.001), whereas there was no significant condition (*p* > 0.43) or interaction (*p* > 0.24) effects. Within each condition, there were no significant differences in biceps brachii EMG activity before TMS and TMES (*p* > 0.08). In addition, no significant repetition (*p* = 0.13), condition (*p* = 0.96) or interaction effects (*p* = 0.25) were observed for triceps rmsEMG. Biceps brachii EMG before MEP (Figure 2) was significantly lower at repetition #9 compared with repetition #1 (*p* = 0.035, *ES* = 1, Δ18.4%), #3 (*p* = 0.14, *ES* = 0.7, Δ12.9%) and #6 (*p* = 0.04, *ES* = 0.5, Δ9.38%), whereas repetition #12 was significantly lower compared with repetition #1 (*p* = 0.03, *ES* = 1, Δ19.38%), #3 (*p* = 0.002, *ES* = 0.7, Δ13.8%) and #6 (*p* = 0.02, *ES* = 0.5, Δ10.3%). Similarly, biceps brachii EMG before CMEP was significantly lower at repetitions #6 (*p* = 0.009, *ES* = 0.6, Δ10.7%), #9 (*p* = 0.02, *ES* = 0.6, Δ13.1%), and #12 (*p* = 0.26, *ES* = 0.5, Δ11%) compared with #1, whereas biceps brachii EMG before M-wave showed lower values at repetitions #6 (*p* = 0.003, *ES* = 0.8, Δ16.7%), #9 (*p* = 0.001, *ES* = 0.9, Δ19%), #12 (*p* = 0.001, *ES* = 1.1, Δ24.7%) compared with repetition #1 and lower values at repetitions #6 (*p* = 0.014, *ES* = 0.5, Δ9.6%), #9 (*p* = 0.015, *ES* = 0.6, Δ12%), #12 (*p* = 0.001, *ES* = 0.8, Δ17.6%) compared with #3 (Figure 2).

### Supraspinal and Spinal Responses

**Figure 3:**
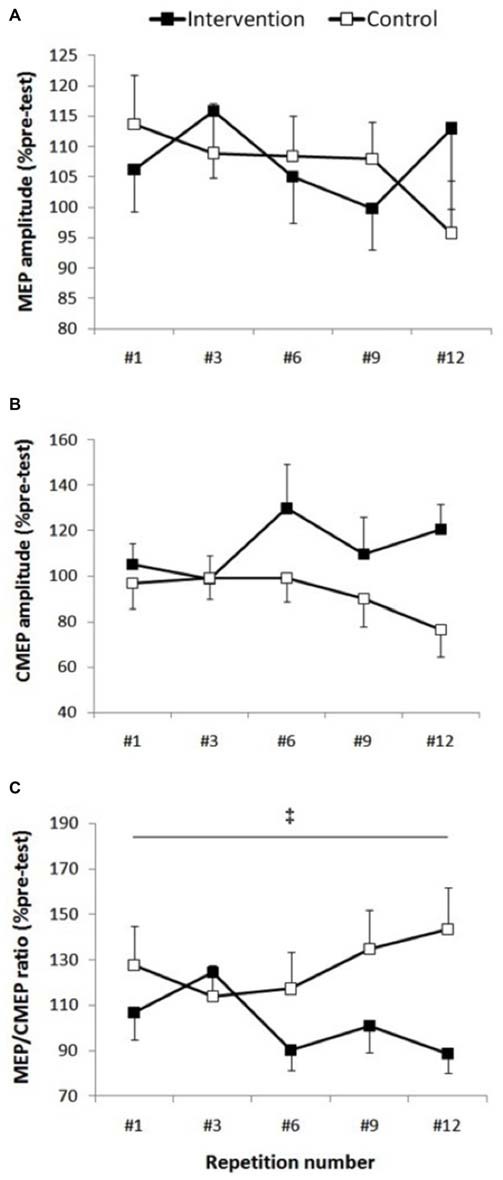
**Corticospinal excitability.** Group data are expressed as means ± SE and normalized to pre-test. **(A)** MEP/M-max normalized to pre-test did not show any condition, repetition or interaction effect (*p* > 0.47). **(B)** There was a trend toward higher cervicomedullary MEP (CMEP) amplitude in the intervention session (*p* = 0.08), however there wasn’t any repetition or interaction effect (*p* > 0.29). **(C)** ^‡^Indicates that MEP/CMEP ratio was significantly smaller in the intervention condition compared with control condition (*p* = 0.042).

MEP peak-to-peak amplitude (Figure 3) showed no significant condition (*F*_(1,13)_ = 0.01, *p* = 0.908), repetitions (*F*_(4,52)_ = 0.61, *p* = 0.655) or interaction effects (*F*_(4,52)_ = 0.90, *p* = 0.471). Similarly, MEP cortical SP did not show any condition (*F*_(1,13)_ = 0.08, *p* = 0.776), repetitions (*F*_(4,52)_ = 1.11, *p* = 0.358) or interaction effects (*F*_(4,52)_ = 0.632, *p* = 0.642).

There was a non-significant trend toward higher CMEP amplitudes (Figure 3) following fatigue intervention compared to control (*F*_(1,13)_ = 3.64, *p* = 0.08, *ES* = 0.5, Δ20.4%), however no repetitions (*F*_(4,52)_ = 0.88, *p* = 0.481) or interaction effects (*F*_(4,52)_ = 1.25, *p* = 0.299) were observed for this measurement.

Although the MEP/CMEP ratio did not show a repetitions effect (*F*_(4,52)_ = 0.39, *p* = 0.810), a significant condition effect (*F*_(1,13)_ = 5.07, *p* = 0.042, *ES* = 2.5, Δ25%) was evident which indicated that the MEP/CMEP ratio was lower in the intervention session (Figure 3). Furthermore, there was no interaction effect for this measure, however there was a strong trend toward significance (*F*_(4,52)_ = 2.42, *p* = 0.06).

### Compound Muscle Action Potential

M-max amplitude recorded during MVCs did not show main effects for condition (*F*_(1,13)_ = 1.18, *p* = 0.296), repetitions (*F*_(4,52)_ = 1.98, *p* = 0.111), or interaction (*F*_(4,52)_ = 0.34, *p* = 0.71).

## Discussion

The main findings of this study were that bilateral knee extensor neuromuscular fatigue decreased dominant EFs force production (large ES), lowered MEP/CMEP ratio (large ES) and showed a trend toward higher CMEP amplitude (medium ES) recorded from biceps brachii. These results suggest that the NLMF observed in the EF muscles could have been mediated by an alteration in excitability of the supraspinal motor circuitries. More specifically, since the EF EMG output (an indication of central motor drive) and responsiveness of spinal motoneurones did not show any decrease following lower limb fatigue, suppression of the motor cortical excitability could have been the main reason for modulation of MEP/CMEP ratio and consequently NLMF.

Exercise-induced fatigue can affect rested, non-exercised muscles and impair their performance (Martin and Rattey, 2007; Kennedy et al., 2013a; Halperin et al., 2014a,b; Kawamoto et al., 2014). Data in the present study support this concept since elbow flexion MVC force was significantly lower (10.3%) following bilateral knee extension fatigue compared with control condition. The protocol used in our study was previously employed by Halperin et al. (2014a) where they demonstrated that the NLMF induced by knee extensors did not affect biceps brachii EMG, but decreased EFs force production, which is in line with our findings. Indeed, most of the NLMF studies that have found decrements in force have also found voluntary activation impairments (knee extensors: Martin and Rattey, 2007; Doix et al., 2013; Halperin et al., 2014b; ankle plantar flexors: Kennedy et al., 2013a; Todd et al., 2003a; Sidhu et al., 2014; and first dorsal interossei: Post et al., 2008), thus suggesting that central fatigue mediated this phenomenon. It has been recommended that an alteration in the responsiveness of corticospinal circuitries might contribute to the inability of CNS to adequately drive non-exercised muscles (Millet and Lepers, 2004). However none of the aforementioned studies directly investigated the role of supraspinal and spinal motoneuron excitability in modulation of central motor drive to the non-exercised muscles.

**Figure 4:**
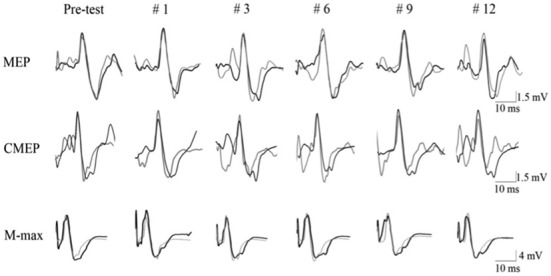
**Evoked raw EMG responses recorded from biceps brachii muscle of a single subject in response to motor cortical (MEP), spinal (CMEP) and peripheral nerve stimulation (M-max) for intervention (dark line) and control (light line) condition.** Data are presented for the pre-test (average of the three pre-test values) and for 5 post-test MVC contractions (repetition #1, #3, #6, #9, #12).

In the present study, the knee extension fatigue resulted in significantly lower MEP/CMEP ratio (25%) and a trend (*p* = 0.08) toward higher CMEP amplitude (20.4%), with no change in MEP size (Figure 4). Initially, the unchanged MEP amplitude following the leg fatigue, might indicate absence of any change in corticospinal pathway innervating the EF muscle; however, a decrease in MEP/CMEP ratio, concomitant with non-significant increase in CMEP, suggest that leg fatigue could have resulted in suppression of excitability in the supraspinal circuitries. Based on this notion, the unchanged MEP and EMG activity during MVCs could be attributed to an increase in central motor drive to maintain the force output. Indeed, previous investigations have shown that increases in cortical motor drive could outweigh the disfacilitation of cortical cell excitability with a net effect of maintaining MEP amplitude (Martin et al., 2006). The notion of increased central motor drive seems to be supported by observing a trend towards higher CMEP amplitude following fatigue intervention where increased supraspinal motor drive could facilitate the spinal motoneurone excitability (Todd et al., 2003b; McNeil et al., 2011). The non-significant but moderate increase in spinal motoneurone responses in the intervention condition could be also be a compensation for the decreased supraspinal excitability (Aboodarda et al., 2015; Nardone et al., 2015) and thereby prevented a drop in the excitability of the corticospinal pathway (i.e., no change in MEP). Millet and Lepers (2004) explains that an increase in spinal excitability could be attributed to decreased presynaptic inhibition and/or fusimotor system facilitation. Nonetheless, our results are in line with Samii et al. (1996) who reported that forearm muscles did not show any changes in MEP after a contralateral homologous fatiguing protocol. Todd et al. (2003b) also found no change in MEP amplitude recorded from the biceps brachii after a contralateral homologous fatiguing protocol. However both of these studies measured the effect of NLMF phenomenon on contralateral homologous muscles. To the best of our knowledge, there are only two published articles that have investigated the effect of lower extremity neuromuscular fatigue on upper limb corticospinal excitability. Takahashi et al. (2011) showed MEP facilitation in the resting first dorsal interosseus and the biceps brachii muscles during and immediately following an intense leg press exercise. Contrary to their findings, Sidhu et al. (2014) reported a depression of MEP amplitude in the EFs after leg cycling task to failure. The reason for the inconsistency between these finding is unclear, however the influence of employing different fatiguing protocols on the activation of type III/IV muscle afferent signaling pathway might explain some of the disparities (further explanation see the next paragraph). Furthermore, neither of these two studies measured spinal excitability separately from the measurement of the corticospinal excitability as a whole. Therefore, it was not known how changes in spinal motoneuron excitability contributed to the changes in MEP amplitude.

It has been suggested that group III/IV muscle afferents from working muscles can cause spinal and supraspinal fatigue to remote muscles (Amann et al., 2013; Sidhu et al., 2014). Indeed, Sidhu et al. (2014) found that in the presence of leg fatigue, group III/IV muscle afferents feedback might contribute to EF supraspinal fatigue. The authors suggested that decreased responsiveness of the motor cortical cells and/or increased intracortical inhibition might be due to the group III/IV-mediated inhibition, or disfacilitation. However, it is unlikely that intracortical inhibition contributed to NLMF effects in present study, since the duration of the cortical SP did not change (Taylor et al., 1996). Pearcey et al. (2014) suggested that the decrease in MEP could be masked by increased spinal excitability. Nonetheless, the impact of these corticospinal excitability changes on force production is unclear. Indeed, Gandevia et al. (1996) found that muscle ischemia impaired voluntary activation while it did not have any effect on measures of corticospinal excitability. Furthermore, Goodall et al. (2012) and Sidhu et al. (2009) reported a significant decrease in voluntary activation after fatiguing exercise, but no change in MEP, suggesting that failure in central motor drive differs from an impaired corticospinal excitability.

The present study had methodological considerations such as participants were instructed to keep the non-fatigued limb inactive (electrical silence) during the fatiguing contractions. Therefore, they might have compromised the maximal muscle force production during fatiguing contractions to avoid mirror activity. In addition, lower force production and no change in biceps brachii EMG in the intervention condition could be due to the non-linear relationship between force and EMG, EMG insensitivity to small force changes and/or a minor shift in wrist pronation-supination, which could influence synergistic muscle contribution (Halperin et al., 2014a).

In conclusion, our data indicate that bilateral knee extensor fatigue produced force deficits in the dominant EFs with no change in biceps brachii EMG activity. Furthermore, we suggested that inhibitory influence of exercise-induced leg fatigue on EFs neuromuscular function could have originated at the level of supraspinal motor circuitries because a trend for a spinal motoneuronal response increase was observed. Moreover, contributions of corticospinal excitability changes to NLMF need to be further investigated, where spinal excitability will be measured independently of the excitability of the whole corticospinal tract, since this will provide insights to the response of different structures to NLMF.

## Author Contributions

All authors listed, have made substantial, direct and intellectual contribution to the work, and approved it for publication.

## Conflict of Interest Statement

The authors declare that the research was conducted in the absence of any commercial or financial relationships that could be construed as a potential conflict of interest.
